# The Effect of Weight Loss and Metabolic Interventions on Recurrence After Atrial Fibrillation Ablation

**DOI:** 10.3390/jcm15166493

**Published:** 2026-08-21

**Authors:** Shihan Fu, Shujie Li, Xiyuan Zhang, Ruoxin Yu, Lin Sun

**Affiliations:** 1 The First Clinical Medical College, Harbin Medical University, Harbin 150081, China; 2022157060@hrbmu.edu.cn (S.F.); 2022152077@hrbmu.edu.cn (S.L.); 2024188007@hrbmu.edu.cn (X.Z.); 2 The Fourth Clinical Medical College, Harbin Medical University, Harbin 150081, China; 2024164029@hrbmu.edu.cn; 3 Department of Cardiology, The First Affiliated Hospital of Harbin Medical University, 23 Youzheng Street, Nangang District, Harbin 150001, China

**Keywords:** atrial fibrillation, catheter ablation, recurrence, obesity, weight loss, SGLT2 inhibitors, GLP-1 receptor agonists

## Abstract

Catheter ablation is the cornerstone of rhythm control in atrial fibrillation (AF), yet recurrence remains common, and obesity is among the most consistently implicated modifiable risk factors. Weight reduction and metabolic pharmacotherapy are increasingly used in the periprocedural period, but whether they act through a shared pathway has not been systematically examined. This narrative review compares the two approaches and asks whether metabolic agents confer protection beyond weight loss itself. Three observations argue that they do not act identically. First, the benefit of weight reduction is dose-dependent yet contingent on delivery: a structured, physician-led risk-factor program reduced 12-month arrhythmia recurrence (risk ratio 0.53), whereas nurse-led care that improved guideline adherence without structured delivery did not alter the primary endpoint. Second, sodium-glucose cotransporter 2 inhibitors (SGLT2i) have been associated with reduced recurrence across BMI strata despite producing only modest weight loss; notably, a randomized trial in patients without cardiovascular or metabolic comorbidity showed no additional benefit, whereas benefit was observed in patients with type 2 diabetes and heart failure. Third, glucagon-like peptide-1 receptor agonists (GLP-1RA) achieve greater weight loss but yield inconsistent recurrence data, and Mendelian randomization suggests their cardiometabolic benefit is largely BMI-mediated, whereas that of SGLT2i is weight-independent. Together, these observations are consistent with a working hypothesis of two partly distinct atrial substrates—an obesity-related substrate responsive to weight reduction, and a metabolic-inflammatory substrate that may respond to SGLT2i predominantly when metabolic comorbidity is present. This framework is hypothesis-generating: it rests on indirect, cross-study comparisons and has not been tested by formal mediation analysis. If confirmed, it would imply that the two interventions are complementary rather than interchangeable.

## 1. Introduction

**Figure 1:**
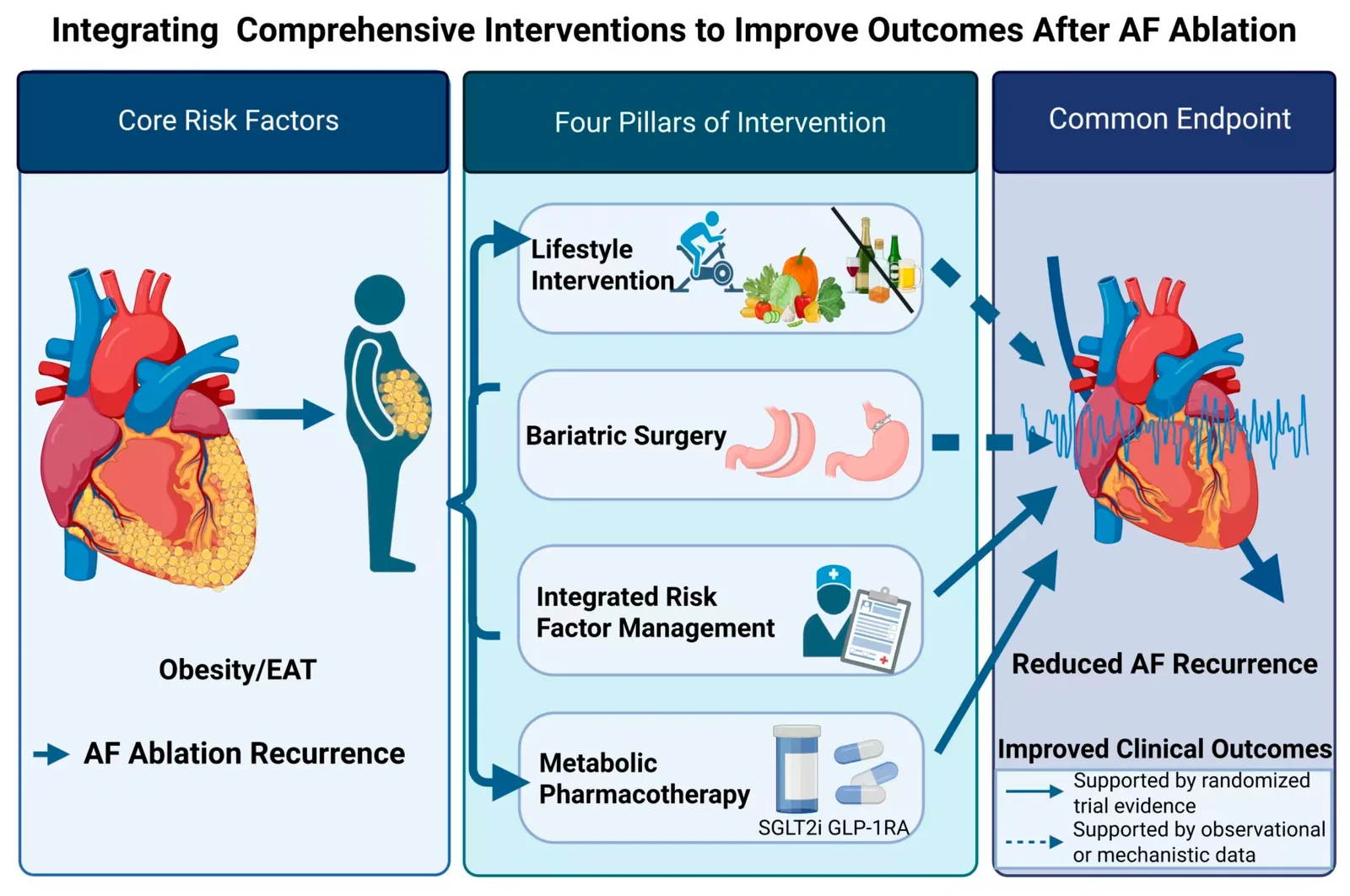
A framework for comprehensive management to reduce recurrence after atrial fibrillation (AF) ablation. The left panel identifies core, modifiable contributors to post-ablation recurrence—obesity and epicardial adipose tissue (EAT). The central panel groups interventions into four categories: (1) lifestyle intervention (exercise, dietary modification, alcohol restriction); (2) bariatric surgery; (3) integrated risk-factor management; and (4) metabolic pharmacotherapy (SGLT2i and GLP-1RA). These interventions are supported by evidence of differing strength—from randomized trials to observational and mechanistic data—as detailed in Table 1; solid arrows denote interventions supported by randomized evidence and dashed arrows those resting on observational or mechanistic data. The right panel indicates the shared goal of reduced AF recurrence and improved clinical outcomes. The figure summarizes proposed relationships and is not intended to imply equivalent certainty across interventions. Abbreviations: AF: atrial fibrillation; EAT: Epicardial Adipose Tissue; GLP-1RA: Glucagon-Like Peptide-1 Receptor Agonist; SGLT2i: Sodium-Glucose Cotransporter 2 Inhibitor. Created in BioRender. Xiao, L. (2026) https://BioRender.com/q42fy7y.

Atrial fibrillation (AF) is the most common sustained arrhythmia, and its incidence and prevalence are on the rise worldwide. Approximately 50 million people worldwide have AF, and its prevalence is projected to double over the next 35 years. AF increases the risk of stroke, thromboembolism, heart failure, and death [1]. AF is also associated with obesity, high blood pressure, obstructive sleep apnea, excessive alcohol consumption, and smoking [2]. The two main treatment strategies for AF are rate control and rhythm control. Rate control involves using medications that prolong the atrioventricular node refractory period to slow the ventricular rate. In contrast, rhythm control involves using antiarrhythmic drugs, electrical cardioversion, or ablation to restore and maintain sinus rhythm [3]. Pulmonary veins were initially believed to be ectopic trigger sites for AF; as a result, pulmonary vein isolation (PVI) has become the most widely used treatment for AF today [4]. Studies have shown that, for patients with symptomatic AF, catheter ablation is more effective than antiarrhythmic drugs as an initial rhythm control strategy [5]. However, the recurrence of AF following catheter ablation remains a major challenge for the electrophysiology community [6]. Recurrence remains common; approximately one in eight patients undergoes a repeat AF ablation within one year [7]. Because many recurrences are managed medically or not re-treated, repeat ablation is a procedural endpoint that underestimates the true recurrence rate. Active management of modifiable risk factors, supplemented by selective pharmacotherapy, appears to improve the success rate of ablation. The molecular mechanisms underlying AF recurrence after catheter ablation primarily include TGF-β1- and MMP-mediated structural remodeling and fibrosis, ion channel dysfunction, inflammatory responses, autonomic nervous system imbalance, and genetic and epigenetic alterations [8]. The primary strategies for preventing recurrence of AF after ablation include antiarrhythmic medications and lifestyle interventions. Among these, lifestyle interventions are widely recommended because they are simple to implement and cost-effective. Obesity is an independent risk factor for recurrence of AF after ablation [9,10,11]. This review focuses on the role of weight loss and metabolic interventions in preventing AF recurrence. Figure 1 illustrates the logical flow of this paper.

Several recent reviews have summarized lifestyle modification or SGLT2i in AF, but these are organized around interventions and treat weight loss and metabolic pharmacotherapy as parallel components of risk-factor management [12,13,14]. The present review is instead organized around the substrate. We ask a single question—whether metabolic agents act through weight reduction or independently of it—and use the discordant trials as the informative cases rather than as noise: the neutral result of dapagliflozin in metabolically healthy candidates, the neutral result of pre-ablation GLP-1RA exposure, and the divergent outcomes of two structurally different risk-factor programs.

PubMed and Web of Science were searched from January 2010 to June 2026 using combinations of “atrial fibrillation”, “catheter ablation”, “recurrence”, “obesity”, “weight loss”, “bariatric surgery”, “SGLT2 inhibitor” and “GLP-1 receptor agonist”. English-language clinical studies, meta-analyses and mechanistic investigations relevant to post-ablation recurrence were included; case reports and conference abstracts were excluded. Reference lists of retrieved articles were screened for additional sources. As this is a narrative review, studies were selected on relevance rather than by predefined criteria, and formal risk-of-bias assessment was not performed. Evidence was weighted by design: randomized trials were given precedence over observational and propensity-matched cohorts, which in turn were weighted above uncontrolled or cross-sectional data; mechanistic findings from animal or non-atrial human tissue were treated as supporting biological plausibility only, and not as evidence that an intervention reduces post-ablation recurrence. Where studies disagreed, we gave greater weight to randomized data and to studies using continuous rhythm monitoring. As a narrative review, the search was not intended to be systematic and no formal risk-of-bias assessment was performed; the synthesis should not be read as a comprehensive or quantitative appraisal.

**Table 1:** Summary of clinical studies evaluating weight-loss and metabolic interventions and atrial fibrillation recurrence after catheter ablation.

Ref.	Design/Evidence Level	Population & Metabolic Phenotype	Intervention	Timing vs. Ablation	*n*	Monitoring/Recurrence Definition	Follow-Up	Main Result	Principal Limitation
[15]	RCT, open-label, multicenter	Nonpermanent symptomatic AF, BMI ≥ 27, ≥1 risk factor, first ablation	LRFM vs. usual care	At catheter ablation	122	Freedom from AF in 12-month period after ablation	12 mo	HR 0.53 for recurrence vs. UC	Single-center; open-label
[16]	RCT	Post-ablation AF, paroxysmal AF	Nurse-led lifestyle intervention vs. standard care	Post-ablation	160	Primary: SF-36 quality of life; Secondary: AF recurrence, complications	12 mo	Lower recurrence & complications; better function	Single-center; 12-mo long-term results need further validation
[17]	RCT	AF	Nurse-guided care vs. standard care	Outpatient chronic care	1375	Composite of cardiovascular death, stroke, bleeding, or acute coronary syndrome	37 mo	Better guideline adherence but no difference in primary endpoint	Delivery/team experience may confound; single-center
[18]	RCT	Post-ablation AF	APN-led follow-up	Post-ablation	65	AF recurrence, patient knowledge, lifestyle, satisfaction	6 mo	Lower recurrence; lower alcohol intake	Short 6-mo follow-up; single-center; small sample
[19]	Observational cohort	AF patients undergoing catheter ablation, categorized by BMI (normal < 25.0, overweight 25.0–29.9, obese ≥ 30.0)	BMI as exposure	—	3333	AF recurrence at 12 months following catheter ablation	12 mo	Obese vs. overweight: HR = 1.223	Registry-based; BMI alone cannot distinguish fat distribution
[20]	Systematic review & meta-analysis	Obese AF patients undergoing catheter ablation (CA) with weight-loss interventions	Weight loss (>10% vs. <10%)	Post-ablation	1851	AF recurrence/NR	Variable	Overall weight loss did not significantly reduce recurrence (RR = 0.76, 95% CI 0.49–1.18, *p* = 0.22); however, at >12-month follow-up, recurrence was significantly reduced with weight loss (RR = 0.47, *p* < 0.0001), regardless of magnitude (>10% or <10%).	Predominantly observational studies; heterogeneity and potential bias; variable follow-up durations across included studies
[21]	Systematic review & meta-analysis	Patients undergoing ablative therapy for AF	weight loss	Pre-ablation	1425	AF recurrence/NR	Variable	Overall weight loss significantly reduced recurrence: RR = 0.35 (95% CI 0.18–0.67);	Predominantly observational studies; heterogeneity and potential bias
[22]	Retrospective observational cohort	Morbidly obese, AF ablation	Pre-ablation bariatric surgery	Pre-ablation	239	AF recurrence/NR	36 mo	Lower recurrence than no surgery	Selection; residual confounding
[23]	Observational	Morbidly obese	Bariatric surgery	Pre-ablation	255	Arrhythmia recurrence	Mean 29 ± 13 months post-ablation	BS significantly reduced BMI, HbA1c, and SBP, all *p* < 0.0001; no procedural complications in BS group	Selection bias
[24]	RCT, Prospective, open-label	AF, first ablation, no CV/metabolic comorbidity	Dapagliflozin 10 mg/day vs. placebo	Post-ablation	200	Early recurrence after catheter ablation	3 mo	Neutral—no additional recurrence reduction	Off-indication population; power
[25]	Observational	AF + T2DM	SGLT2i inhibitor	Peri-procedural	182	Post-operative AF recurrence	Variable	SGLT2i associated with decreased risk of recurrence; aHR = 0.65	Non-randomized
[26]	Prospective observational	AF + HF, no T2DM	SGLT2i, periprocedural	Periprocedural	102	Catheter ablation outcomes	12 mo	SGLT2i improved ablation outcomes in HF patients without T2DM	Non-randomized
[27]	Observational	AF + HF	SGLT2i	Post-ablation	736	AF recurrence after 3-mo blanking	1315 person-years	AF recurrence: 22.6% vs. 35.8%; aHR = 0.56; composite CV outcome aHR = 0.58	Non-randomized
[28]	Observational	AF	SGLT2i	Post-ablation	1146	AF recurrence/NR	20.5 ± 13.7 months	SGLT2i 17.5% vs. non-SGLT2i 29.3%, HR = 0.59 (95% CI 0.46–0.75, *p* < 0.001), with consistent benefit across MetS status and BMI spectrum	Non-randomized
[29]	Meta-analysis	AF + T2DM	SGLT2i vs. all antidiabetics/DPP-4i	—	1043	AF recurrence/NR	Variable	SGLT2i vs. all antidiabetic drugs: HR = 0.57 (95% CI 0.44–0.73); SGLT2i vs. DPP-4i: HR = 0.41 (95% CI 0.24–0.70)	Predominantly observational studies
[30]	RCT	AF + T2DM	Tofogliflozin vs. alogliptin	—	80	AF recurrence after catheter ablation	12 mo	Tofogliflozin superior	Small sample size; single-center
[31]	Cohort (EHR)	Pre-ablation GLP-1RA users	GLP-1RA	Pre-ablation	3250	Composite: cardioversion/new AAD/repeat ablation	12 mo	Neutral (HR 1.04, 95% CI 0.92–1.19)	Retrospective database study with residual confounding
[32]	Single-center PSM	Obese, post-ablation	Semaglutide	Peri-ablation	362	AF recurrence/NR	18 mo	Significant weight reduction with semaglutide	Single-center; weight vs. drug effect not separable
[33]	Retrospective	Obese AF ablation	GLP-1RA	Baseline (pre-ablation)	3116	Primary composite endpoint: cardioversion, new AAD, or redo AF ablation	12 mo	GLP-1 RA associated with significantly lower risk of the primary composite endpoint (HR = 0.72, 95% CI 0.65–0.80, *p* < 0.001); mortality HR = 0.61 (*p* < 0.001);	Retrospective
[34]	Prospective cohort	AF + T2DM	SGLT2i vs. GLP-1RA vs. combination	Post-ablation	482	Implantable continuous monitoring	12 mo	Combination therapy significantly reduced the composite endpoint (HR = 0.443) with concomitant reduction in inflammatory/oxidative stress markers.	T2DM only; weight contribution not isolated

Studies are grouped by intervention and ordered by level of evidence. Because recurrence ascertainment differed across studies (monitoring modality, blanking period, qualifying-episode duration, and binary recurrence vs. AF burden), effect estimates are not directly comparable between rows; see Section 5.2. Where the primary study did not specify the rhythm-monitoring modality, this is marked as “not reported (NR)”; most studies reported binary recurrence rather than AF burden. For meta-analyses, “*n*” denotes the pooled sample size. Abbreviations: AAD, antiarrhythmic drug; AF, atrial fibrillation; APN, advanced practice nurse; BMI, body mass index; EHR, electronic health record; GLP-1RA, glucagon-like peptide-1 receptor agonist; HF, heart failure; HR, hazard ratio; LRFM, lifestyle and risk-factor management; NR, not reported; PSM, propensity-score matched; RCT, randomized controlled trial; SF-36, 36-item Short-Form Health Survey; SGLT2i, sodium–glucose cotransporter 2 inhibitor; T2DM, type 2 diabetes mellitus.

## 2. The Relationship Between Obesity and AF Recurrence

### 2.1. Mechanistic Basis of the Obesity Substrate

Numerous epidemiological studies have shown that the risk of AF increases with weight gain. The Framingham Heart Study reported that for every one-unit increase in body mass index, the risk of AF increases by 4% [35]. Obesity not only increases the risk of AF but is also significantly associated with the recurrence of AF following treatment. Studies have shown that, compared with overweight patients, patients with a body mass index (BMI) of ≥30 kg/m^2^ are 1.2 times more likely to experience a recurrence of AF during a 12-month follow-up period [19]. This has prompted clinicians to place great emphasis on weight management for patients with AF.

Nevertheless, studies on the “obesity paradox”—which show that patients with AF who are overweight or obese have a reduced risk of cardiovascular and all-cause mortality, as well as a lower risk of major bleeding compared to their normal-weight peers—call for clinicians to adopt a more meticulous and individualized approach to weight management for patients with AF [36]. Studies have found that among patients with AF who undergo catheter ablation, the risk of AF recurrence is reduced regardless of whether weight loss exceeds 10% or is less than 10% [20]. However, the benefits are more pronounced in patients who have lost ≥10% of their body weight, have a history of AF of less than 12 months, and have experienced weight loss before ablation [21]. A statement from the American Heart Association recommends that, for patients with AF, the initial weight-loss goal be 10%, and the ultimate target body mass index be below 25 kg/m^2^ [35,37].

**Figure 2:**
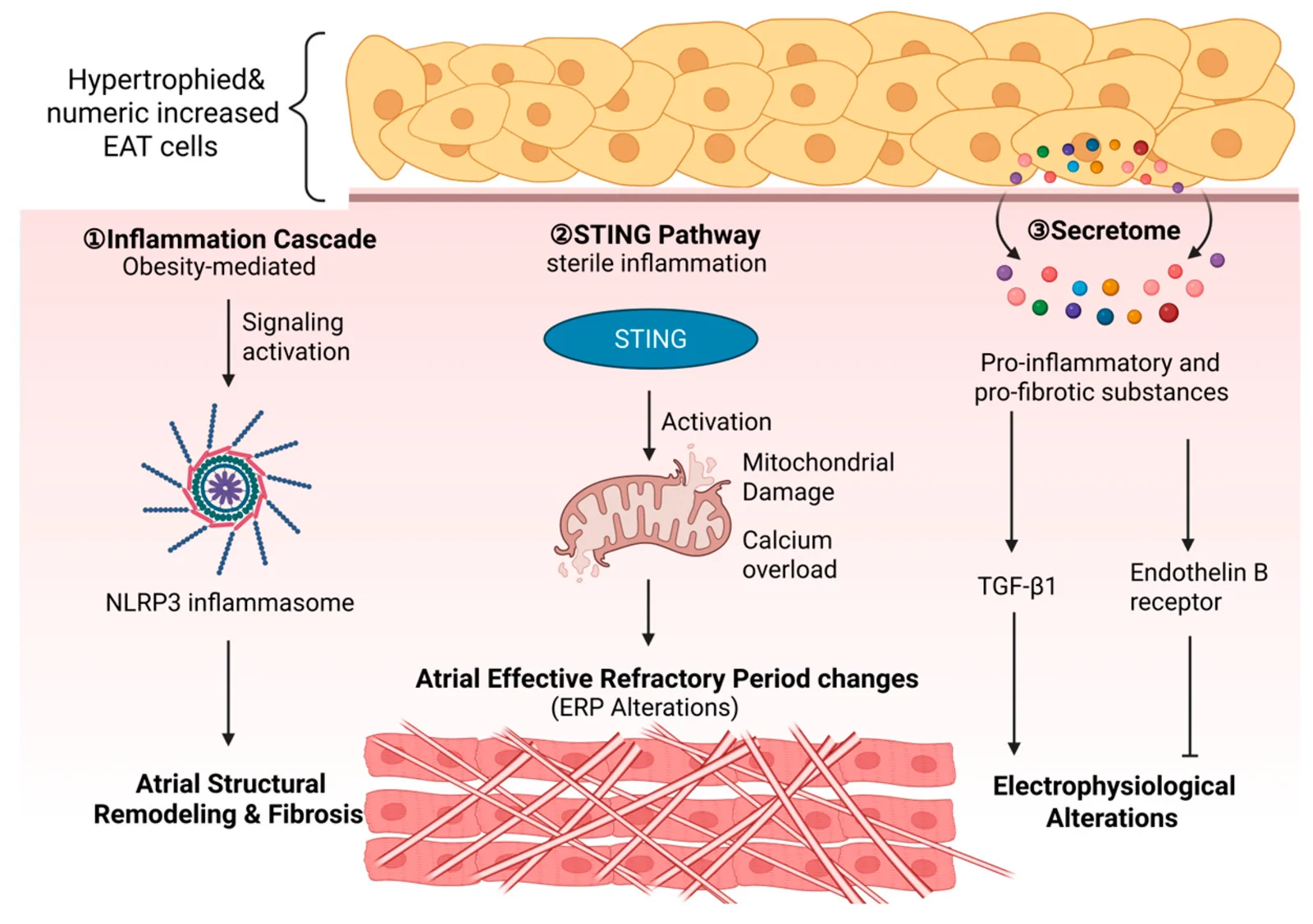
Pathophysiological Mechanisms of Epicardial Adipose Tissue (EAT)-Mediated Atrial Remodeling. This schematic illustrates the proposed pathways through which hypertrophied and numerically increased EAT cells contribute to the pathogenesis of AF. The detrimental cross-talk between EAT and the underlying atrial myocardium is driven by three primary mechanisms: (1) Inflammation Cascade: Obesity-mediated signaling activates the NLRP3 inflammasome, which directly promotes atrial structural remodeling and fibrosis. (2) STING Pathway: The activation of STING triggers sterile inflammation, leading to mitochondrial damage and calcium overload. These intracellular disruptions cause significant alterations in the Atrial Effective Refractory Period (ERP). (3) Secretome: EAT functions as an active endocrine organ, releasing a variety of pro-inflammatory and pro-fibrotic substances (such as TGF-β1) and interacting with pathways like the Endothelin B receptor. This paracrine signaling ultimately results in profound electrophysiological alterations in the atrial tissue. Arrows (→) indicate activation or promotion; blunt-ended arrows (⊣) indicate inhibition. Colours are used for illustrative purposes only and do not convey additional meaning. Abbreviations: AF: Atrial fibrillation; EAT: Epicardial Adipose Tissue; ERP: Effective Refractory Period; NLRP3: NOD-, LRR- and pyrin domain-containing protein 3; STING: Stimulator of Interferon Genes; TGF-β1: Transforming Growth Factor-beta 1. Created in BioRender. Xiao, L. (2026) https://BioRender.com/c26t03q.

It is worth noting that some research teams have found that both being overweight and underweight increase the risk of AF recurrence [38]. This suggests that using BMI to assess obesity has limitations, as it cannot distinguish adipose from lean tissue, potentially leading to incorrect classification of body fat in the study population and introducing bias. There are several reasons why obesity contributes to the recurrence of AF. Epicardial adipose tissue (EAT) is a type of visceral fat located between the myocardium and the visceral pericardium. The volume of epicardial adipose tissue is closely associated with the occurrence, severity, and recurrence of AF. An increase in left atrial EAT volume is associated with increased left atrial fibrosis and dilation [39]. Atrial fibrosis and dilation are important predictors of AF recurrence and progression [36]. Epicardial adipose tissue may be associated with the re-entry mechanism of AF [40]. At the same time, epicardial adipose tissue can also secrete substances that promote inflammation, fibrosis, and arrhythmias [41]. The relationship between inflammation, fibrosis, oxidative stress, and AF has been well established. Inflammatory cytokines increase oxidative stress, collagen deposition, and abnormal calcium metabolism, leading to tissue damage, localized fibrosis, and electrophysiological changes in atrial tissue [42]. Patients with obesity and AF may exhibit an increase in EAT volume. Furthermore, increased NLRP3 inflammasome activity mediated by obesity also promotes the development of AF [43]. An animal study found that the stimulator of interferon genes (STING) serves as a central hub in intracellular damage and inflammatory cascades, and that sterile inflammation is a common pathological feature of obesity and AF. STING gene knockout alleviates obesity-induced mitochondrial calcium overload and mitochondrial damage, thereby significantly prolonging the effective refractory period of the atria in obese rats and reducing obesity-induced atrial remodeling [44]. The relevant mechanisms are illustrated in Figure 2. Metabolic syndrome is increasingly recognized as a driver of atrial cardiomyopathy—a substrate of structural, electrical, and inflammatory atrial remodeling—that may constitute the pathway through which metabolic dysfunction promotes AF and its recurrence [45].

### 2.2. The Obesity Paradox Reconsidered

Reports that higher body mass index (BMI) is associated with better outcomes after ablation have been used to question whether weight reduction should be pursued at all. Several considerations suggest that this inference is unsafe. First, BMI does not distinguish fat mass from lean mass, nor does it distinguish visceral from subcutaneous distribution. The expected benefit of weight loss is further modified by disease stage, the degree of atrial remodeling, visceral (rather than total) adiposity, age, and the timing of the intervention [39,40], and two individuals with identical body mass index may carry substantially different atrial substrates. Second, studies of post-ablation outcomes condition on having already undergone ablation. Patients are selected for the procedure based on expected benefit, so obese patients who reach the laboratory represent a more favorably selected group than their non-obese counterparts. Conditioning on this shared consequence of exposure and prognosis can induce an apparent protective association where none exists [46]. Third, unintentional weight loss accompanies heart failure, malignancy and frailty. Cross-sectional comparisons that do not distinguish intentional from unintentional weight loss will attribute the poor prognosis associated with illness-related weight loss to low body mass itself [47]. Fourth, the phenotype rather than the quantity of adiposity may be decisive. Even metabolically healthy obesity carries increased cardiovascular risk through persistent low-grade inflammation, which is itself shaped by sex and fat distribution [48].

Against these observations stands direct interventional evidence. In a controlled ovine model, weight reduction produced a graded reversal of the atrial substrate, with attenuation of fibrosis, fatty infiltration, transforming growth factor-β1 expression, and connexin 43 remodeling [49]. A dose-dependent structural response of this kind is difficult to reconcile with a genuinely protective effect of adiposity. The paradox is therefore better understood as an artifact of measurement and selection than as a biological phenomenon, and it does not provide grounds for withholding weight reduction from obese candidates for ablation.

## 3. Traditional Strategies for Weight and Risk Factor Management

Weight-loss-associated reductions in the expression of transforming growth factor-β1 and endothelin B receptors can promote collagen resorption and reverse myocardial fibrosis, thereby attenuating the electrophysiological and structural remodeling caused by obesity [49]. Patients who have lost weight have a lower probability of developing acute kidney injury, acute respiratory distress syndrome, and infections during their hospital stay [50]. Multiple studies have shown that weight loss can benefit patients with AF.

### 3.1. Lifestyle Interventions

Physical activity and diet are both direct factors influencing body weight. Some studies suggest that a U-shaped curve can represent the relationship between physical activity and the risk of AF—people who meet the exercise recommendations outlined in the guidelines have a significantly reduced risk of AF. In contrast, those who are physically inactive or engage in excessive exercise do not experience this benefit [51]. A study found that the AF recurrence rate was higher in the group with slowed heart rate recovery than in the group with normal heart rate recovery [52]. This association is consistent with, but does not by itself establish, an effect of physical inactivity or short-term overexertion on recurrence risk. For elite athletes, long-term participation in intense endurance exercise increases the risk of AF [53]. A 6-month exercise intervention trial found that exercise intervention can reduce the recurrence rate of arrhythmias and improve symptom severity in patients with AF; here, “exercise intervention” refers to aerobic exercise performed at home and under supervision [54]. Interestingly, a study on factors influencing the recurrence of exercise-induced AF after ablation found that recurrence rates were the same for athletes and non-athletes, and that resuming physical activity after the procedure had no significant effect on recurrence of AF 1 year or more post-procedure [55]. However, other studies have shown that engaging in high-intensity physical activity after AF ablation can reduce the incidence of serious adverse events [56]. Differences in research findings may be attributed to varying criteria for assessing exercise intensity, differences in patients’ baseline characteristics, and variations in follow-up duration. This suggests that future studies need to quantify “moderate” and “high-intensity” exercise more precisely, rather than making general recommendations about exercise. Although the relationship between exercise and the risk of AF recurrence is difficult to conclude definitively, moderate physical activity can help achieve weight loss. Whether exercise should be initiated during or after the blanking period, and at what intensity, has not been addressed by any dedicated trial; current recommendations are extrapolated from non-ablation populations.

The Mediterranean diet generally refers to the traditional dietary patterns of coastal countries such as Greece and Italy. It is plant-based, prioritizes healthy fats such as extra-virgin olive oil, encourages consumption of fish and moderate amounts of dairy products, and limits red meat and processed foods. This dietary pattern not only effectively promotes weight loss and reduces waist circumference but also significantly reduces visceral fat [57]. PREDIMED is a multicenter, randomized, primary prevention trial that demonstrates that the Mediterranean diet can serve as a sustainable and ideal model for preventing cardiovascular disease [58]. A study analyzing the relationship between adherence to the Mediterranean diet and EAT in patients with AF found that higher adherence to the Mediterranean diet was associated with lower EAT levels. Although the study did not find a significant association between EAT ≥ 135 g and the risk of recurrence of atrial arrhythmias after ablation, it did find a significant association with persistent AF. Although the study results were negative, the research still suggests that following the Mediterranean diet offers certain benefits [59]. In addition, alcohol can also affect the recurrence of AF. Alcohol consumption can alter the body’s substrate oxidation pathways, causing it to prioritize the metabolism of alcohol metabolites while inhibiting lipid oxidation, thereby promoting the accumulation of visceral fat [60,61]. Encouraging patients to limit their alcohol intake to less than 20 g per week after ablation can reduce the risk of AF recurrence [62]. Moderate exercise, following a Mediterranean diet, and reducing alcohol intake can all lead to weight loss; however, whether these lifestyle changes reduce the risk of AF recurrence indirectly by affecting body weight or act via a direct mechanism on the atria remains to be further investigated. Although the exact mechanisms are not yet clear, there is sufficient clinical evidence that adopting these lifestyle changes reduces the risk of AF recurrence. This further underscores the importance of lifestyle interventions for patients following ablation procedures.

### 3.2. Bariatric Surgery

Bariatric surgery achieves weight loss of a magnitude that lifestyle intervention rarely attains, and is therefore the setting in which any dose-dependent effect on the atrial substrate should be most apparent. In morbidly obese patients undergoing AF ablation, those who had undergone bariatric surgery before the procedure had lower arrhythmia recurrence than those who had not [22]. Outcomes in morbidly obese patients after bariatric surgery approached those of patients without morbid obesity [23]. Pre-ablation weight loss achieved through metabolic bariatric surgery was associated with shorter hospital stays and lower rates of acute kidney injury, respiratory complications, and infections, without differences in cardiac, pericardial, or bleeding complications [50]. This is relevant because concern about operative risk is a common reason not to pursue surgical weight loss before ablation. The magnitude of weight loss after surgery corresponds to the range over which graded reversal of atrial substrate was demonstrated experimentally [49], providing a plausible mechanistic account rather than an empirical association alone.

All available data are observational, and patients selected for bariatric surgery differ systematically from those not selected. Weight loss after surgery is neither uniform nor permanent, and no study has examined whether recurrence rises with weight regain. The interval between surgery and ablation has not been standardized, although the reverse remodeling on which the benefit depends is unlikely to be complete within a few months. For patients with morbid obesity in whom ablation is contemplated, current observational evidence may support considering bariatric surgery before, rather than after, ablation in selected morbidly obese patients, although this cannot be generalized and the optimal interval remains undefined.

Because many morbidly obese candidates for ablation carry substantial cardiovascular and anesthetic risk, less-invasive options are of interest. Endovascular bariatric approaches, in particular transcatheter gastric artery embolization, have been described as a minimally invasive strategy with relatively short recovery and potential utility in patients at high surgical risk; the evidence remains preliminary, and randomized studies with clinically meaningful outcomes are still required [63].

### 3.3. Comorbidity-Directed Management (MS/OSA)

Metabolic syndrome (MS) and obstructive sleep apnea (OSA) are recognized as independent risk factors for AF recurrence [64]. A subgroup analysis of AF patients who underwent ablation therapy revealed that the recurrence rate among patients with both MS and OSA was significantly higher than among those with MS or OSA alone [65]. Obstructive sleep apnea has been associated with an approximately twofold increase in the risk of incident AF in a large polysomnography-based cohort. However, this association was significant only among patients under 65 years of age [66]. Evidence from community-based cohorts has been less consistent, with one study finding that central, rather than obstructive, sleep apnea predicts incident AF [67]. Whether treating obstructive sleep apnea alters post-ablation outcome follows a pattern that parallels the risk-factor programs discussed below. Observational cohorts and their pooled analyses consistently report lower recurrence among patients using continuous positive airway pressure than among untreated patients with obstructive sleep apnea [68,69]. A randomized trial of continuous positive airway pressure after pulmonary vein isolation, however, did not demonstrate a reduction in recurrence [70]. The discrepancy is plausibly attributable to adherence: observational comparisons contrast patients who use the therapy with those who do not, whereas randomization to an intervention with well-documented compliance problems dilutes any true effect. As with structured versus unstructured risk-factor programs, the determinant may be delivery rather than the intervention itself.

### 3.4. Comprehensive Risk Factor Management Program

A study of Asian patients with AF demonstrated that following the ABC pathway (“A” for preventing stroke through anticoagulation; “B” for better symptom control; “C” for management of cardiovascular risk factors and comorbidities) reduces the risk of adverse outcomes [71]. Compared with standard care, nurse-led lifestyle interventions help improve patients’ physical function, role limitations, vitality, overall health, and social functioning. The nurse-led lifestyle intervention group also had significantly lower rates of AF recurrence and complications than the standard care group [16]. It is worth noting that another randomized controlled trial found that although guideline adherence was significantly higher in the nurse-guided group than in the standard-care group, there was no difference in the risk of the primary endpoint between the two groups [17]. This may suggest that nurse-led care provided by an experienced team is what offers clinical benefits. In the ARREST-AF trial, patients were randomized to either the Lifestyle and Risk Factor Management (LRFM) group or the Usual Care (UC) group. The LRFM group received treatment in a structured, physician-led, personalized outpatient setting aimed at reducing modifiable risk factors. By comparing the proportion of patients who remained free of AF 12 months after ablation, the study found that the LRFM group had a risk ratio of 0.53 for arrhythmia recurrence within 12 months compared to the UC group. This study further demonstrates that active management of risk factors is crucial for maintaining sinus rhythm following catheter ablation [15]. Another nurse-led trial on post-ablation care for AF demonstrated that follow-up care provided by advanced practice nurses (APNs) to patients after AF ablation significantly reduced the recurrence rate of AF and patients’ alcohol consumption [18]. This underscores the need for comprehensive, structured, and personalized interventions in clinical practice.

The value of improving ablation durability extends beyond the binary recurrence endpoint. If risk-factor modification translates into sustained rhythm control, an individualized question arises as to whether selected patients might eventually be candidates for de-escalation of long-term oral anticoagulation, potentially reducing bleeding burden; this should not be presented as an automatic consequence of successful ablation and requires dedicated study [72]. Effective risk-factor modification may also reduce downstream healthcare utilization—outpatient visits, emergency presentations, hospitalization, cardioversion, antiarrhythmic therapy, and repeat ablation—particularly in obese populations [73].

## 4. Metabolic Drug Interventions

Because effect estimates below derive from studies with markedly different rhythm-monitoring strategies and recurrence definitions (see Section 5.2), apparent differences between therapies should be interpreted with caution.

### 4.1. Molecular Basis of Metabolic Pharmacotherapy in Atrial Remodeling

**Figure 3:**
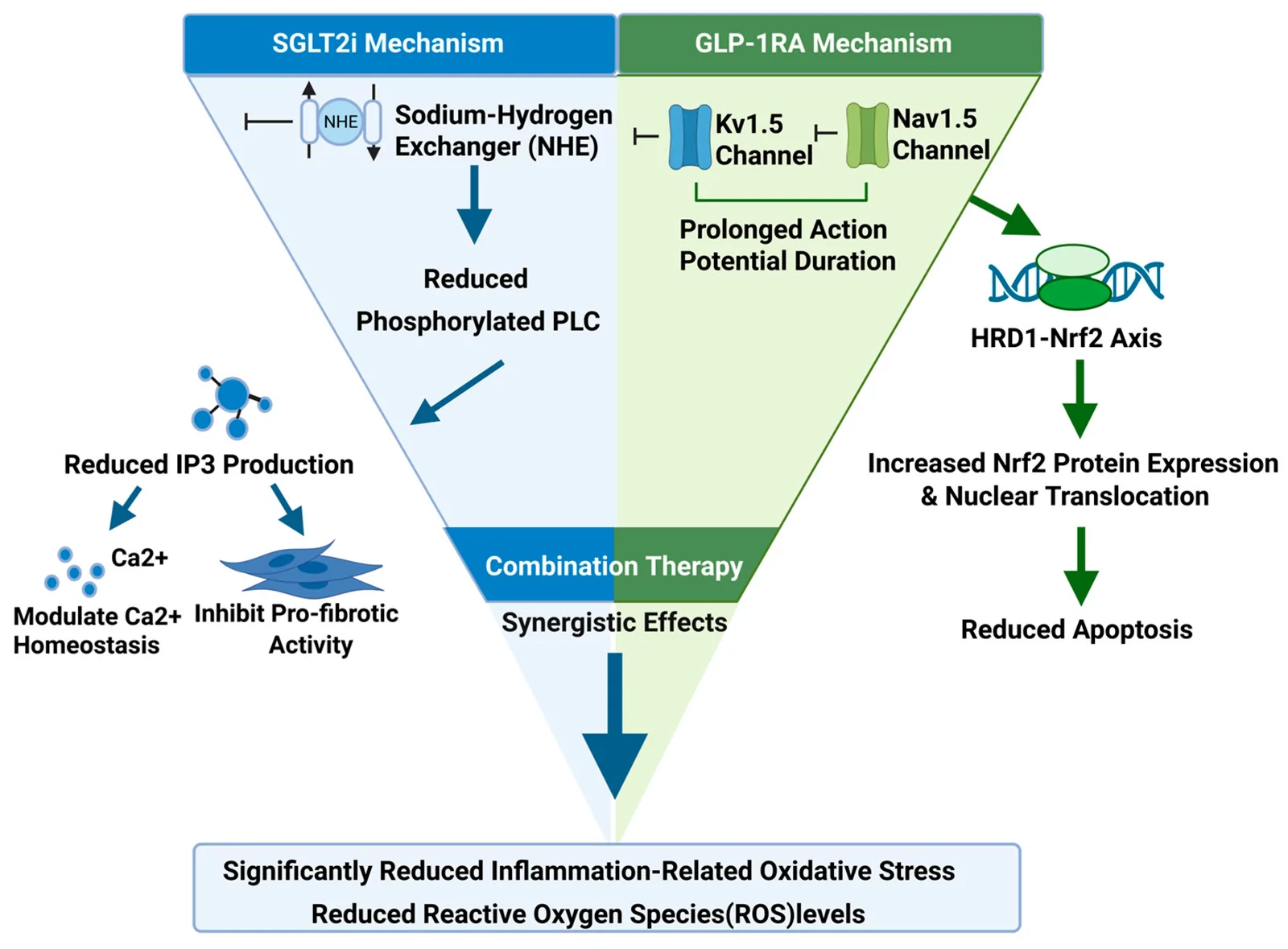
Distinct and Synergistic Mechanisms of SGLT2i and GLP-1RA Combination Therapy. This schematic illustrates the independent intracellular signaling pathways modulated by SGLT2i (**left panel**) and GLP-1 receptor agonists (**right panel**), which are proposed to converge on synergistic cardioprotective effects. The pathways depicted derive largely from preclinical and non-atrial experimental models and remain to be confirmed in the human atrium. The SGLT2i mechanism involves inhibition of the Sodium-Hydrogen Exchanger (NHE), leading to decreased phosphorylation of PLC and a subsequent reduction in IP3 production. This cascade ultimately modulates intracellular Ca^2+^ homeostasis and inhibits pro-fibrotic activity. Concurrently, the GLP-1RA mechanism inhibits Kv1.5 and Nav1.5 channels, thereby prolonging action potential duration. Furthermore, GLP-1RA activates the HRD1-Nrf2 axis, increasing Nrf2 protein expression and nuclear translocation, thereby reducing cellular apoptosis. When used as a Combination Therapy, these distinct pathways yield synergistic effects that significantly attenuate inflammation-related oxidative stress and reduce reactive oxygen species (ROS) levels. Arrows (→) indicate activation or promotion; blunt-ended arrows (⊣) indicate inhibition. Colours are used for illustrative purposes only and do not convey additional meaning. Abbreviations: Ca^2+^: Calcium ion; GLP-1RA: Glucagon-Like Peptide-1 Receptor Agonist; HRD1: HMG-CoA Reductase Degradation Protein 1; IP3: Inositol 1,4,5-trisphosphate; Kv1.5: Voltage-gated Potassium Channel 1.5; Nav1.5: Voltage-gated Sodium Channel 1.5; NHE: Sodium-Hydrogen Exchanger; Nrf2: Nuclear Factor Erythroid 2-Related Factor 2; PLC: Phospholipase C; ROS: Reactive Oxygen Species; SGLT2i: Sodium-Glucose Cotransporter 2 Inhibitor. Created in BioRender. Xiao, L. (2026) https://BioRender.com/2jz0j66.

Before considering clinical outcomes, it is useful to separate the mechanisms by which metabolic agents may act on the atrium, as these mechanisms predict which patients are likely to benefit. The relevant mechanisms are illustrated in Figure 3. The atrial actions of SGLT2i appear largely independent of their glucose-lowering effects. Empagliflozin inhibits the sodium-hydrogen exchanger, reducing phosphorylated phospholipase C expression and inositol trisphosphate generation, thereby restoring calcium homeostasis and attenuating the profibrotic activity of atrial fibroblasts [74]. In an angiotensin II–driven model, dapagliflozin shortened the prolonged action potential duration and restored transient outward, inward rectifier, and late sodium current densities, while limiting left atrial dilatation and interstitial fibrosis [75]. Additional contributions include improved mitochondrial energetics, reduced oxidative stress, and lower circulating levels of C-reactive protein, tumor necrosis factor-α, and interleukin-6 as summarized in Table 2 [76]. Because none of these pathways require a change in body weight, they provide a mechanistic rationale for benefit independent of body mass.

GLP-1 receptor agonists act through a partly distinct set of pathways. Exenatide inhibits human Kv1.5 and Nav1.5 channels and prolongs action potential duration [18]. At the same time, tirzepatide reduces oxidative stress and cardiomyocyte apoptosis by limiting HRD1-mediated ubiquitination and degradation of Nrf2, thereby increasing its nuclear translocation and transcriptional activity [77]. It should be noted that the latter was demonstrated in a doxorubicin cardiotoxicity model rather than in atrial tissue, and extrapolation to the fibrillating atrium remains provisional.

Both drug classes converge on epicardial adipose tissue, which is metabolically active, clinically measurable, and pharmacologically modifiable [78]. In human right atrial appendages, inflamed tissue showed increased oxidative stress and fibrosis, along with upregulated SGLT2 and GLP-1 receptor expression; this pattern was more pronounced in patients with a history of AF [79]. Receptor availability, therefore, appears to be itself a function of the inflammatory substrate. This finding may explain why benefit is observed when metabolic comorbidity is present, but not when it is absent. Mechanistic findings from animal or non-atrial human models support biological plausibility only and cannot establish that an intervention reduces post-ablation recurrence through the proposed pathway.

**Table 2:** Mechanistic evidence for metabolic pharmacotherapy in atrial remodeling.

Agent	Model System	Pathway	Readout	Caveat
SGLT2i (Empagliflozin) [74]	Human atrial fibroblasts; Isoproterenol-induced HF rats	NHE inhibition → ↓ phosphorylated PLC → ↓ IP3 production → ↓ ER Ca^2+^ leakage and extracellular Ca^2+^ entry	↓ fibroblast migration, ↓ collagen I/III, ↓ atrial fibrosis, ↑ LV systolic function	Mechanistic evidence from in vitro and animal HF model; not assessed in post-ablation recurrence trials
GLP-1RA (Exenatide) [75]	HEK293 cells (hKv1.5/hNav1.5); Rat atrial myocytes; Isolated perfused rat hearts; In vivo rat AF model (Ach-CaCl_2_)	Closed-state block of hKv1.5; open-state block of hNav1.5; inhibition of Iss and I_to; prolongation of APD	↓ AF incidence and duration in isolated hearts and in vivo rat model	Preclinical mechanistic and anti-AF susceptibility study; concentrations used (μM) exceed therapeutic plasma levels; not a post-ablation recurrence trial
GIP/GLP-1RA (Tirzepatide) [77]	H9c2 cardiomyocytes; C57BL/6 mice (doxorubicin-induced cardiotoxicity)	Inhibition of HRD1-mediated Nrf2 ubiquitination → ↑ Nrf2 protein stability, nuclear translocation, and transcriptional activity	↓ oxidative stress, ↓ cardiomyocyte apoptosis, ↓ myocardial injury, ↑ cardiac function	Mechanistic study in a chemotherapy-induced cardiotoxicity model (not AF-specific); extrapolation to the fibrillating human atrium requires caution
SGLT2i + GLP-1RA (Empagliflozin + Semaglutide/Liraglutide) [79]	Human right atrial appendage tissue (*n* = 80, with/without AF history); Human coronary artery ECs, AC16 CMs, and PBMCs	Overlapping but distinct cell-specific expression (SGLT2 on ECs/macrophages; GLP-1R on CMs/macrophages); synergistic suppression of inflammation-driven ROS via cAMP/PKA (for GLP-1RA) and SGLT2-related pathways	↓ ROS, ↓ NOX2, ↓ MMP-9, ↓ TGF-β1, ↓ VCAM-1, ↓ p53; restored VASP phosphorylation	Ex vivo human tissue and cell culture evidence; demonstrates mechanistic synergy in atrial substrate, but clinical recurrence endpoints not measured

All findings derive from preclinical (animal, cell, or non-atrial human) models and support biological plausibility only; they do not establish that these agents reduce post-ablation AF recurrence in humans. “→” indicates leads to; “↓” indicates reduction/decrease; “↑” indicates increase. Abbreviations: GIP, glucose-dependent insulinotropic polypeptide; GLP-1RA, glucagon-like peptide-1 receptor agonist; HRD1, HMG-CoA reductase degradation protein 1; NHE, sodium–hydrogen exchanger; Nrf2, nuclear factor erythroid 2–related factor 2; ROS, reactive oxygen species; SGLT2i, sodium–glucose cotransporter 2 inhibitor.

### 4.2. Sodium-Glucose Cotransporter 2 Inhibitors (SGLT2i)

The atrial mechanisms of SGLT2i are summarized in Section 4.1. Clinically, their effect on post-ablation recurrence has been examined across several populations [80]. It should be noted that much of this evidence derives from patients with type 2 diabetes or heart failure rather than from unselected post-ablation cohorts; findings from these broader populations are therefore not directly comparable to studies performed specifically after catheter ablation. An animal study found that empagliflozin improves heart failure and arrhythmias by inhibiting the sodium-hydrogen exchanger (NHE), reducing the expression of phosphorylated PLC and IP3 production, thereby regulating calcium homeostasis and the profibrotic activity of atrial fibroblasts [74]. SGLT2i may also reduce the risk of AF recurrence by decreasing EAT volume, lowering levels of pro-inflammatory factors in circulation (C-reactive protein, tumor necrosis factor-α, and interleukin-6), improving myocardial energy metabolism, and reducing oxidative stress [76,78].

The DARE-AF trial, which evaluated dapagliflozin in patients with AF scheduled to undergo their first catheter ablation for a condition for which the drug had no prior indication, found that, in the absence of cardiovascular and metabolic comorbidities, SGLT2i therapy did not further reduce the rate of AF recurrence beyond the reduction in atrial remodeling associated with these comorbidities [24]. However, in patients with type 2 diabetes, the use of SGLT2i is associated with a significant reduction in the risk of AF recurrence [25]. In patients with AF who do not have T2DM but do have heart failure, periprocedural use of SGLT2i may also improve the efficacy of catheter ablation [26]. In patients with heart failure, the use of SGLT2i is associated with a reduced risk of AF recurrence and a reduced risk of the composite endpoint of cardiovascular death, thromboembolic events, or cardiovascular hospitalizations following catheter ablation for AF [27,81]. The benefits for patients with heart failure may be related to the fact that SGLT2i can significantly improve cardiac mechanical function and left atrial compliance [82].

The effect of SGLT2i on AF recurrence was similar across the BMI ranges examined [28]. It has been suggested that the benefits of SGLT2i may not be affected by BMI; however, this requires confirmation through prospective studies. It is worth noting that, regarding the risk of ischemic stroke in patients with AF following ablation, some studies have found no difference in the incidence of ischemic stroke regardless of whether SGLT2i were used [83]. However, some studies have also found that the use of SGLT2i can reduce the risk of ischemic stroke and lower the rate of hospital readmission following AF catheter ablation [84]. A Kaplan–Meier curve based on imputed data showed that, compared with all antidiabetic drugs (log-rank test, *p* = 0.00011) and dipeptidyl peptidase-4 inhibitors (DPP-4i) (log-rank test, *p* = 0.01), the SGLT2i group had a significantly lower risk of AF recurrence [29]. These findings suggest an association between SGLT2 inhibitor use and lower AF recurrence, although residual confounding cannot be excluded in this non-randomized comparison. Furthermore, a prospective study has confirmed that tofogliflozin is more effective than alogliptin in reducing the recurrence of AF after catheter ablation [30].

### 4.3. Glucagon-like Peptide-1 Receptor Agonists (GLP-1RAs)

The atrial mechanisms of GLP-1 receptor agonists are summarized in Section 4.1. The clinical recurrence data are less consistent than for SGLT2i [75]. Like SGLT2i, GLP-1RAs can reduce the risk of AF by decreasing left atrial volume [78]. An animal study demonstrated that the GLP-1RA tirzepatide exerts direct cardioprotective effects, which are primarily mediated by the HRD1-dependent transcription factor Nrf2. By reducing the ubiquitination and degradation of Nrf2, the drug increases its protein levels, nuclear translocation, and transcriptional activity, thereby reducing oxidative stress and cardiomyocyte apoptosis [77]. A cohort study examining the relationship between pre-ablation use of GLP-1RA and subsequent AF recurrence showed that pre-ablation use of GLP-1RA was not associated with a reduced risk of post-ablation cardioversion, new antiarrhythmic drug therapy, or repeat AF ablation (HR: 1.04 [95% CI: 0.92–1.19]) [31]. Another single-center, propensity-matched study found that treatment with semaglutide reduces the risk of AF recurrence in obese patients following catheter ablation for AF, suggesting its potential as an adjunctive therapy [32]. Among these, the pre-ablation GLP-1RA cohort [31] and the semaglutide propensity-matched study [32] were performed specifically in post-ablation patients, whereas other estimates derive from broader AF or obesity populations; this distinction should be borne in mind when comparing effect sizes. The difference between the two may be that patients with AF in the latter group are obese, making it difficult to determine whether the effects of GLP-1RA are independent or result from their action on obesity, a risk factor for AF recurrence. Some studies have found that a reduction in BMI primarily mediates the cardiovascular and metabolic benefits of GLP-1RA, whereas SGLT2i provides independent protective effects [85]. However, another retrospective study found that the effect of GLP-1RA in reducing AF recurrence in obese patients was no different between those with a BMI > 40 and those with a BMI ≤ 40 [33]. Numerous clinical trials have demonstrated that SGLT2i and GLP-1RAs in patients with AF who have undergone catheter ablation can reduce the risk of AF recurrence and offer multiple benefits, including lower rates of hospitalization and cardiovascular adverse events. Several of these observational analyses derive from overlapping electronic health record networks; concordant findings therefore provide limited independent replication and may share the same sources of confounding. However, the exact mechanisms underlying these effects and the benefits in patients without metabolic comorbidities still require further confirmation.

### 4.4. Periprocedural Considerations for GLP-1RA

The rapid adoption of GLP-1 receptor agonists raises practical questions specific to the periprocedural period that efficacy data do not address.

Delayed gastric emptying and sedation. GLP-1 receptor agonists slow gastric emptying, and retained gastric contents have been reported in treated patients despite adherence to standard fasting intervals. Because AF ablation is performed under deep sedation or general anesthesia, often with transoesophageal or intracardiac echocardiography, this is directly relevant to the risk of aspiration. Early perioperative practice tended to favor withholding these agents before elective procedures [86]. More recent multisociety guidance has moved from routine discontinuation toward an individualized, risk-stratified approach based on shared decision-making among the proceduralist, anaesthesia, and prescribing teams, weighing the metabolic need for the agent against individual aspiration risk; a higher-risk profile (dose-escalation phase, higher doses, and gastrointestinal symptoms) may warrant a pre-procedure clear-liquid diet or temporary interruption, whereas routine cessation is no longer advised for all patients [87]. Although these documents address surgical and endoscopic procedures rather than catheter ablation specifically, the principles apply directly to AF ablation, which is performed under deep sedation or general anaesthesia, and electrophysiology teams should incorporate them into pre-procedure planning.

Weight loss achieved pharmacologically includes a substantial proportion of lean mass [88]. This is relevant to the present review in two ways: it reinforces the inadequacy of body mass index as a measure of the atrial substrate (Section 2.2), and it raises the possibility that fat-mass reduction and lean-mass loss have opposing consequences that offset one another in trials using weight as the exposure. Weight is substantially regained after cessation [89]. If benefit is mediated by adiposity reduction, it is unlikely to persist beyond treatment, and the appropriate duration of therapy around ablation is entirely undefined. Trials have used exposures ranging from established pre-ablation therapy to initiation after the procedure, and the observation that pre-ablation exposure was not associated with reduced recurrence may reflect insufficient duration before the index procedure rather than the absence of effect [31]. Whether these agents should be started before ablation, and how long before, is unresolved. These considerations do not argue against the use of GLP-1 receptor agonists in obese candidates for ablation. Still, they indicate that their periprocedural management requires explicit protocols rather than extrapolation from metabolic practice.

### 4.5. Combination Therapy

Because SGLT2i and GLP-1 receptor agonists act on partially non-overlapping pathways, their combination is mechanistically attractive rather than merely additive in terms of convenience. Direct evidence for interaction at the atrial level comes from human right atrial appendage tissue. In 80 specimens obtained during cardiac surgery, inflamed appendages exhibited increased oxidative stress and fibrosis, alongside upregulated SGLT2 and GLP-1 receptor expression, both of which were more pronounced in patients with a history of AF. Exposure to either agent reduced reactive oxygen species, and the combination was more effective than either alone [79]. This provides a tissue-level rationale for combination therapy that does not depend on additional weight loss. Clinically, a prospective cohort of patients with type 2 diabetes undergoing AF ablation was stratified into SGLT2 inhibitor, GLP-1 receptor agonist, and combination groups, with recurrence assessed over 12 months using implantable continuous monitoring [34]. Combination therapy was associated with significantly lower recurrence and with lower circulating inflammatory and oxidative stress markers than either monotherapy, despite the combination group having poorer glycemic control at baseline. That benefit accrued in the arm with the higher glycated hemoglobin is itself informative, since it dissociates the atrial effect from glycemic improvement. The use of continuous monitoring is notable, as it reduces much of the ascertainment variability that limits comparisons across the wider literature.

Two qualifications apply. The available evidence is confined to patients with type 2 diabetes, so whether combination therapy benefits non-diabetic obese candidates is untested. And no study has yet separated the contribution of each agent from that of the greater weight loss achieved on combination therapy—precisely the mediation question raised throughout this review. Combination therapy is therefore best regarded at present as a reasonable option in diabetic patients with substantial metabolic burden, rather than as a generalizable strategy.

## 5. Ablation-Specific Considerations

### 5.1. EAT and the Post-Ablation Atrium

Epicardial adipose tissue is the anatomical interface through which obesity acts upon the atrium. It is in direct contact with atrial myocardium without an intervening fascial plane, and its secretory products therefore reach the myocardium by paracrine diffusion [90,91]. Extracellular vesicles isolated from epicardial fat of patients with AF carry proinflammatory, profibrotic, and proarrhythmic cargo, and induce atrial myopathy in experimental systems [41].

Imaging evidence indicates that this relationship is spatially organized rather than diffuse. In a study combining Dixon sequences for adipose quantification with late gadolinium enhancement for fibrosis in patients with AF, left atrial epicardial adipose tissue was associated with both left atrial volume and fibrosis burden, and the distance between fibrotic regions and the nearest adipose tissue was analyzed to assess colocalization [40]. Adherence to a Mediterranean dietary pattern is associated with lower epicardial adipose volume in patients with AF [58], and both SGLT2i and GLP-1 receptor agonists modulate this depot [78].

Three questions specific to ablation remain unanswered. First, does ablation itself alter epicardial adipose tissue? Thermal or electrical injury is delivered immediately adjacent to this depot, and any acute inflammatory response within it could plausibly contribute to early recurrence during the blanking period. Second, does the spatial relationship between adipose tissue and ablation lesions influence lesion durability? Fat is a poor conductor, yet its distribution is not accounted for in current lesion-set design. Third, is a reduction in epicardial adipose volume, rather than total body weight, the proximate mediator of benefit from weight loss and metabolic therapy?

None of these has been addressed prospectively. Serial epicardial adipose imaging before and after ablation would be technically straightforward and would test a mediator that is more mechanistically proximate than body mass index.

### 5.2. Heterogeneity in Recurrence Ascertainment

Comparison of effect sizes across the studies summarized above is constrained by a methodological problem that is rarely stated explicitly: recurrence is not measured the same way twice. Detection depends on monitoring intensity. Intermittent Holter recording, symptom-triggered event monitoring, serial short-term recording, and implantable continuous monitoring differ by several fold in the proportion of arrhythmias they capture, and asymptomatic episodes—the majority in many post-ablation cohorts—are detected only by continuous or near-continuous methods [92]. A study reporting a low recurrence rate with intermittent monitoring and another reporting a high rate with implantable monitoring may describe the same patients.

Definitions vary in three further respects. The blanking period is variously set at thirty, sixty, or ninety days, and early recurrence within this window is by convention disregarded despite being a recognized predictor of late recurrence [93]. The minimum qualifying duration is usually 30 s, but it is not universal. And composite endpoints incorporating repeat ablation or new antiarrhythmic drug initiation—as used in at least one of the GLP-1 receptor agonist cohorts discussed above—reflect clinical decision-making as much as they do arrhythmia. Apparent inconsistency between studies may reflect ascertainment rather than biology, and this is a plausible partial explanation for the discordant GLP-1 receptor agonist literature. And binary recurrence discards information: AF burden is more sensitive to substrate modification than a dichotomous endpoint, and the dose-dependent benefit of weight reduction was demonstrated most clearly using burden rather than recurrence. Studies in this field should therefore report monitoring modality, monitoring duration, blanking period, and qualifying episode length as standard, and readers should treat cross-study comparisons of recurrence rates with corresponding caution [94]. Finally, the evidence summarized here largely predates the widespread adoption of pulsed-field ablation (PFA). Because PFA alters lesion formation and may change durability and recurrence patterns, the effect sizes discussed above will require re-estimation in PFA-treated populations.

## 6. Limitations

This review has several limitations. It is narrative rather than systematic; although the search strategy is stated (in the introduction), studies were selected on relevance rather than by predefined criteria, and formal risk-of-bias assessment was not undertaken. The evidence base is dominated by observational studies, single-center cohorts and secondary analyses, with few randomized trials designed with arrhythmia recurrence as the primary endpoint. Effect sizes across studies are not directly comparable due to heterogeneity in recurrence ascertainment, as discussed in Section 5.2. Several of the mechanistic findings cited derive from animal or non-atrial models, and their extrapolation to the human atrium is provisional. Finally, the central proposition of this review—that metabolic agents act partly independently of weight reduction—is supported by indirect evidence, including consistency of effect across body mass index strata and the divergent results of trials in patients with and without metabolic comorbidity, but has not been tested by formal mediation analysis in any published study.

## 7. Controversies and Future Directions

The central question raised by this review—whether metabolic agents act through weight reduction or independently of it—cannot be settled by subgroup analysis. The two-substrate model should be regarded as a testable hypothesis: prospective studies incorporating formal mediation analysis, with weight change and inflammatory/metabolic markers as candidate mediators, are needed to determine whether SGLT2i exert weight-independent atrial effects and whether GLP-1RA benefit is predominantly weight-mediated. Trials should prespecify change in body weight as a mediator, with epicardial adipose volume as a secondary mediator, to estimate direct and indirect effects. Two-step Mendelian randomization using cis-acting variants in drug target genes, anchored to both glycated hemoglobin and body mass index, provides a complementary approach; such designs have been applied to cardio-renal-metabolic endpoints but not to post-ablation recurrence [85]. Until monitoring intensity and recurrence definitions are standardized, effect sizes cannot be meaningfully pooled (Section 5.2). Continuous monitoring should be the reference standard in trials of substrate-directed therapy, and AF burden should be reported alongside binary recurrence [94]. The neutral result of dapagliflozin in candidates without cardiometabolic comorbidity raises the question of whether any metabolic agent benefits this group [24]. This is the population in which weight reduction alone may be sufficient, and in which pharmacotherapy currently lacks justification.

Adequately powered trials of GLP-1 receptor agonists with arrhythmia endpoints are needed. Existing data are observational and discordant [31]. Given the scale of current prescribing, a dedicated trial with rhythm outcomes is warranted. As lesion durability improves, the relative contribution of modifiable substrate to recurrence may increase; the effect sizes reported here require re-estimation in the pulsed-field era [95].

## 8. Conclusions

Obesity and metabolic dysfunction are among the few genuinely modifiable determinants of recurrence after AF ablation; however, the evidence reviewed here suggests that weight reduction and metabolic pharmacotherapy do not act on the same target. Weight loss appears to modify an obesity-related atrial substrate—epicardial adipose accumulation, mechanical stretch and fibrosis—and its benefit is dose-dependent but realized only when the intervention is structured and sustained. SGLT2i may act on a metabolic-inflammatory substrate and appear to exert clinically relevant effects that are at least partly independent of body weight; in the single randomized trial performed in candidates without cardiometabolic comorbidity, no additional benefit was observed. GLP-1RAs occupy an intermediate position, with the benefit that current evidence primarily attributes to weight reduction. These interventions are therefore complementary rather than interchangeable, and these observations raise the hypothesis—not yet established—that patient selection might ultimately be informed by metabolic phenotype in addition to body mass index. Two limitations temper these conclusions: no study has formally tested weight change as a mediator, and heterogeneity in rhythm monitoring and recurrence definitions constrains cross-study comparison. Trials that prespecify mediation analysis and harmonize recurrence ascertainment are the necessary next step.

## Data Availability

Data sharing does not apply to this article, as no datasets were generated or analyzed during the current study.

## Abbreviations

The following abbreviations are used in this manuscript:

AADs	Antiarrhythmic drugs
AF	Atrial fibrillation
APNs	Advanced practice nurses
BMI	Body mass index
CI	Confidence interval
DPP-4i	Dipeptidyl peptidase-4 inhibitors
EAT	Epicardial adipose tissue
eGFR	Estimated glomerular filtration rate
GLP-1RA	Glucagon-like peptide-1 receptor agonist
HR	Hazard ratio
HRD1	HMG-CoA reductase degradation protein 1
IP3	Inositol 1,4,5-trisphosphate
LRFM	Lifestyle and Risk Factor Management
MHO	Metabolically healthy obesity
MMP	Matrix metalloproteinase
MS	Metabolic syndrome
NHE	Sodium–hydrogen exchanger
NLRP3	NOD-, LRR- and pyrin domain-containing protein 3
Nrf2	Nuclear factor erythroid 2-related factor 2
OSA	Obstructive sleep apnea
PLC	Phospholipase C
PVI	Pulmonary vein isolation
RCT	Randomized controlled trial
SGLT2i	Sodium-glucose cotransporter 2 inhibitor
STING	Stimulator of interferon genes
T2DM	Type 2 diabetes mellitus
TGF-β1	Transforming growth factor-β1
UC	Usual care
